# Effects of Excess Brain-Derived Human α-Synuclein on Synaptic Vesicle Trafficking

**DOI:** 10.3389/fnins.2021.639414

**Published:** 2021-02-04

**Authors:** Cristina Román-Vendrell, Audrey T. Medeiros, John B. Sanderson, Haiyang Jiang, Tim Bartels, Jennifer R. Morgan

**Affiliations:** ^1^The Eugene Bell Center for Regenerative Biology and Tissue Engineering, Marine Biological Laboratory, Woods Hole, MA, United States; ^2^Harvard Medical School, Brigham and Women’s Hospital, Boston, MA, United States; ^3^UK Dementia Research Institute, University College London, London, United Kingdom

**Keywords:** clathrin mediated endocytosis, electron microscopy, endosome, lamprey, reticulospinal synapse

## Abstract

α-Synuclein is a presynaptic protein that regulates synaptic vesicle trafficking under physiological conditions. However, in several neurodegenerative diseases, including Parkinson’s disease, dementia with Lewy bodies, and multiple system atrophy, α-synuclein accumulates throughout the neuron, including at synapses, leading to altered synaptic function, neurotoxicity, and motor, cognitive, and autonomic dysfunction. Neurons typically contain both monomeric and multimeric forms of α-synuclein, and it is generally accepted that disrupting the balance between them promotes aggregation and neurotoxicity. However, it remains unclear how distinct molecular species of α-synuclein affect synapses where α-synuclein is normally expressed. Using the lamprey reticulospinal synapse model, we previously showed that acute introduction of excess recombinant monomeric or dimeric α-synuclein impaired distinct stages of clathrin-mediated synaptic vesicle endocytosis, leading to a loss of synaptic vesicles. Here, we expand this knowledge by investigating the effects of native, physiological α-synuclein isolated from the brain of a neuropathologically normal human subject, which comprised predominantly helically folded multimeric α-synuclein with a minor component of monomeric α-synuclein. After acute introduction of excess brain-derived human α-synuclein, there was a moderate reduction in the synaptic vesicle cluster and an increase in the number of large, atypical vesicles called “cisternae.” In addition, brain-derived α-synuclein increased synaptic vesicle and cisternae sizes and induced atypical fusion/fission events at the active zone. In contrast to monomeric or dimeric α-synuclein, the brain-derived multimeric α-synuclein did not appear to alter clathrin-mediated synaptic vesicle endocytosis. Taken together, these data suggest that excess brain-derived human α-synuclein impairs intracellular vesicle trafficking and further corroborate the idea that different molecular species of α-synuclein produce distinct trafficking defects at synapses. These findings provide insights into the mechanisms by which excess α-synuclein contributes to synaptic deficits and disease phenotypes.

## Introduction

Synucleinopathies, including Parkinson’s disease, dementia with Lewy Bodies (DLB) and multiple system atrophy, are neurodegenerative diseases that are pathologically characterized by the abnormal accumulation and aggregation of α-synuclein within neuronal cell bodies and neurites (Spillantini and Goedert, 2000; Henderson et al., 2019; Sulzer and Edwards, 2019; Brás et al., 2020). The role of α-synuclein as a key pathogenic protein in these diseases has prompted researchers to better understand its normal function and pathophysiology. While the physiological function of α-synuclein is still incompletely understood, it localizes to presynaptic terminals (Maroteaux et al., 1988) where it interacts with VAMP and synapsin (Burre et al., 2010; Atias et al., 2019), regulates synaptic vesicle pool size (Murphy et al., 2000), and plays a role in both exocytosis and endocytosis of synaptic vesicles (Vargas et al., 2014, 2017; Logan et al., 2017). Synaptic accumulation of α-synuclein may be an early pathological event in synucleinopathies, leading to disruptions of axonal endosomal trafficking, as well as synaptic dysfunction (Nemani et al., 2010; Scott et al., 2010; Boassa et al., 2013; Eisbach and Outeiro, 2013; Spinelli et al., 2014; Volpicelli-Daley et al., 2014; Hunn et al., 2015). Furthermore, synaptic aggregation of α-synuclein is highly correlated with cognitive deficits and dementia in DLB patients (Kramer and Schulz-Schaeffer, 2007; Schulz-Schaeffer, 2010). These observations highlight the importance of understanding the direct effects of excess α-synuclein at synapses.

Decades of biochemical research have suggested that α-synuclein exists predominantly as a soluble, unfolded monomer that folds into an alpha-helical structure when presented with small lipid vesicles, such as synaptic vesicles (Davidson et al., 1998; Chandra et al., 2003; Diao et al., 2013). However, recent studies have provided strong evidence that native, physiological α-synuclein exists as helically folded multimers, principally as tetramers and related multimers (Bartels et al., 2011; Wang et al., 2011; Dettmer et al., 2013; Xu et al., 2019). Moreover, different molecular species of α-synuclein (e.g., monomers, dimers, tetramers, hexamers) appear to exist in equilibrium, and altering this equilibrium leads to modified membrane associations and aggregation, synaptic dysfunction and neurotoxicity (Dettmer et al., 2015a, 2017; Nuber et al., 2018; Fanning et al., 2020). Novel purification and cross-linking methods from human brain and mammalian cells have provided biochemical insight into the composition of native, physiological α-synuclein, which comprises 60, 80, and 100 kDa oligomers of α-synuclein (consistent with tetramers, hexamers, and octomers, respectively), as well as monomeric α-synuclein at 14 kDa (Bartels et al., 2011; Dettmer et al., 2013; Luth et al., 2015). However, the precise effects of different molecular species of α-synuclein on neuronal function, including synaptic vesicle trafficking, remain unclear.

Using the lamprey giant reticulospinal synapse as a model, our lab previously showed that acute introduction of excess recombinant monomeric or dimeric human α-synuclein led to a loss of synaptic vesicles, expansion of the plasma membrane, and increased numbers of clathrin-coated structures and other endocytic intermediates, indicating impaired synaptic vesicle recycling (Busch et al., 2014; Medeiros et al., 2017; Banks et al., 2020). An inhibition of endocytosis, but not exocytosis, was also reported at mammalian calyx of Held synapses dialyzed with recombinant human α-synuclein (Xu et al., 2016; Eguchi et al., 2017). Interestingly, at lamprey synapses where clathrin-mediated endocytosis is the dominant mode of synaptic vesicle endocytosis, monomeric and dimeric α-synuclein inhibited different stages of the process (Busch et al., 2014; Medeiros et al., 2017, 2018; Banks et al., 2020). While monomeric α-synuclein impaired the uncoating of clathrin-coated vesicles (CCVs), dimeric α-synuclein impaired an earlier stage of vesicle fission (Medeiros et al., 2017, 2018; Banks et al., 2020). Together, these studies demonstrated that different molecular species of α-synuclein produce distinct effects on synaptic vesicle endocytosis. In this study we focused on α-synuclein purified from the brain of a neuropathologically normal human subject, comprising helically folded multimers of α-synuclein (60, 80, and 100 kDa species) with a minor component of monomeric α-synuclein (14 kDa). When introduced acutely to lamprey synapses, brain-derived human α-synuclein reduced the synaptic vesicle cluster and altered synaptic vesicle morphology consistent with intracellular trafficking defects, but without obvious effects on synaptic vesicle endocytosis from the plasma membrane. These data provide additional evidence that different molecular species of α-synuclein can produce distinct effects on synaptic vesicle trafficking and suggest that α-synuclein multimers may be somewhat protective against the endocytic defects observed with other molecular species of α-synuclein.

## Materials and Methods

### Isolation and Purification of α-Synuclein From Human Brain Tissue

The brain sample used for purifying α-synuclein was provided by the Newcastle Brain Tissue Resource (Newcastle upon Tyne, United Kingdom). This sample was isolated from the cingulate gyrus (cortex) of a healthy control individual [Caucasian female, age 74, post-mortem interval 53 h. Pathology: Braak 0, McKeith none, CERAD (neuritic plaques) none, Braak NFT (TAU) 3]. Consent was obtained from the patient prior to death at the brain collection center. The brain bank approved of the proposal for the use of human tissue in this study, and the IRB at TB’s institution deemed the planned use of this tissue to be appropriate and ethical.

Tissue pieces weighing ∼500 mg were Dounce homogenized with 20 strokes at 2500 rpm in four volumes (weight:volume) Tris-buffered saline (TBS) (20 mM Tris–HCl, 500 mM NaCl, pH 7.5) with a complete protease inhibitor tablet (Sigma-Aldrich, St. Louis, MO, United States). Homogenates were centrifuged for 5 min at 1000 × *g* at 4°C to remove highly insoluble structures and tissue debris. The resulting supernatants were centrifuged for 30 min at 175,000 × *g*. The high-speed supernatant was collected, flash-frozen in liquid nitrogen, and stored at 80°C until fractionation. After thawing, the supernatants were fractionated by size and buffer-exchanged into 50 mM ammonium acetate (pH 7.40) using size exclusion chromatography (SEC) with a Superose 12 10/300GL Increase column (General Electric, Boston, MA, United States). Crosslinked samples were run on an 4–12% Bis-Tris gel to determine the molecular species in each sample. The fraction containing the highest level of α-synuclein, as determined by ELISA (SEC fraction 12, corresponding to a molecular weight of ∼60–80 kDa), was split into aliquots and either cross-linked with disuccinimidyl glutarate (DSG) for multimer analysis by Western blotting or left in the native form for axonal microinjections, as described below. Another aliquot of the same sample was immunodepleted of α-synuclein using a monoclonal antibody (Anti-α-Synuclein, clone 2F12, MABN1817 Sigma-Aldrich) for use as a negative control in the synapse experiments.

### Circular Dichroism Spectroscopy

Crosslinked α-synuclein for structural assessment was purified as above and additionally immunoprecipitated using the Pierce Direct IP Kit (Thermo Fisher Scientific, Waltham, MA, United States), according to the manufacturer’s instructions. Antibody 2F12 was used as a capture antibody (300 μg per reaction). Volumes of wash and incubation buffers were adapted to the total volume input of the sample. Elution fractions were further concentrated using Amicon concentration columns (Millipore, Burlington, MA, United States) according to the manufacturer’s instructions. The fractions were checked for the purity of α-synuclein multimer using immunoblotting and Coomassie staining. Approximately 10 μM α-synuclein samples were added to a 1 mm path length quartz cuvette for far-UV CD and analyzed using J-1500 CD spectrometer (JASCO) at 25°C. Buffer (50 mM ammonium acetate, pH 7.4) spectra were recorded and subtracted.

### Microinjections and Stimulation

**FIGURE 1:**
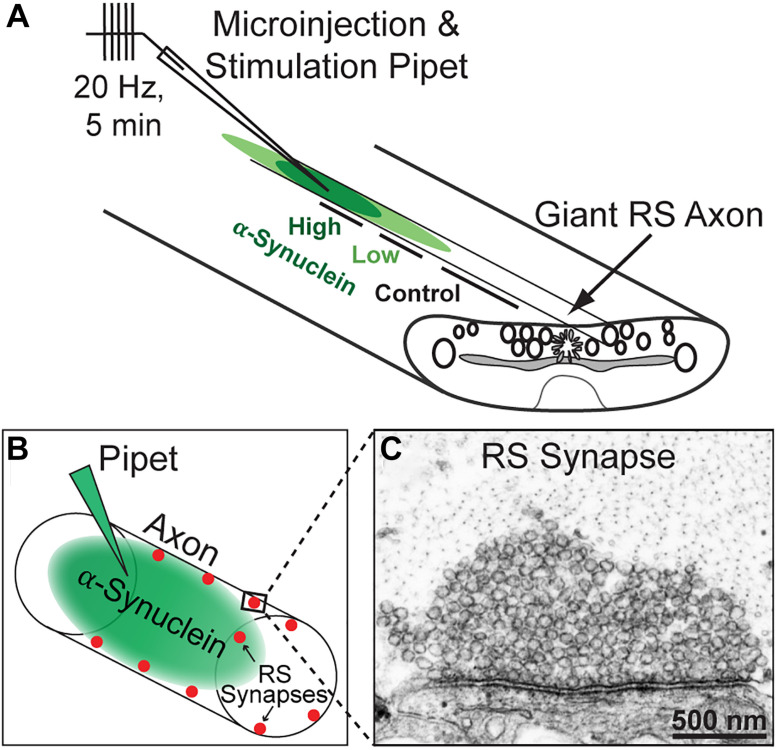
Lamprey reticulospinal (RS) synapses and microinjection strategy. **(A)** Diagram of a lamprey spinal cord showing axonal microinjection and stimulation strategy. Brain-derived human α-synuclein was co-injected into RS axons along with a fluorescent dye of similar molecular weight. After stimulation and fixation, synapses were evaluated within three regions of the axon: those treated with higher concentrations of the protein (estimated 40–80 nM based on the fluorescence of co-injected dye), lower concentrations of the protein (4–39 nM), or no protein (0 nM; Controls). **(B)** Diagram of an injected axon showing the locations of RS synapses along the axonal perimeter. **(C)** Electron micrograph of an unstimulated, control RS synapse showing the large size of the synaptic vesicle cluster.

All animal procedures were approved by the Institutional Animal Care and Use Committee at the Marine Biological Laboratory in Woods Hole, MA following standards set by the National Institutes of Health. Lampreys (*Petromyzon marinus*; 11–13 cm; 5–7 years old of either sex) were anesthetized in 0.1 g/L MS-222 (tricaine methanesulfonate; Syndel, Ferndale, WA, United States). Next, 2–3 cm segments of spinal cord were dissected and pinned ventral side up in a Sylgard-lined dish containing fresh, oxygenated Lamprey Ringer (100 mM NaCl, 2.1 mM KCl, 1.8 mM MgCl_2_, 4 mM glucose, 2 mM HEPES, 0.5 mM L-glutamine, 2.6 mM CaCl_2_, pH 7.4). Axonal microinjections were performed as previously described (Medeiros et al., 2017; Walsh et al., 2018; Banks et al., 2020; Soll et al., 2020). Briefly, SEC fraction 12 containing human α-synuclein was diluted in lamprey internal solution (180 mM KCl and 10 mM HEPES K+; pH 7.4) to a pipet concentration of 800 nM and subsequently loaded into glass microelectrodes (20–25 MΩ) for microinjection into giant RS axons. Microinjections were performed using small pulses of nitrogen (4–20 ms, 40 psi, 0.2 Hz) delivered through a picospritzer. Co-injection with a fluorescent dye (70 KDa fluorescein dextran; Thermo Fisher) approximating the molecular weight of brain-derived α-synuclein multimers allowed us to determine the spread of the protein along the axon with respect to the injection site. After injection, the proteins are normally diluted ∼10–20 times near the injection site and up to 200 times farther away. We therefore estimated that the human α-synuclein was diluted to a final axonal concentration between 40 and 80 nM at distances near to the injection site (30–125 μm), which we refer to as the “high concentration”; we also estimated the α-synuclein concentration to be ∼4–30 nM at distances farther away from the injection site (140–400 μm), which we refer to as the “low concentration” (Figure 1A). Control synapses were collected from the same axon at distances >430 μm from the injection site where no protein had diffused, providing an internal control. Immunodepleted samples were injected using the same methods. After injections, the axons were stimulated at 20 Hz for 5 min, as in our prior studies (Busch et al., 2014; Medeiros et al., 2017; Banks et al., 2020; Soll et al., 2020).

### Electron Microscopy and Imaging

Following stimulation, spinal cords were immediately fixed in placein 3% glutaraldehyde with 2% paraformaldehyde in 0.1 M Na cacodylate, pH 7.4, and processed for electron microscopy (EM) as previously described (Walsh et al., 2018). Spinal cords were thin sectioned at 70 nm and counterstained with 2% uranyl acetate followed by 0.4% lead citrate. Images of individual synapses within the injected axon were acquired using a JEOL JEM CX transmission electron microscope at 37,000× or 59,000× magnification. For each experimental condition, EM images from *n* = 21–37 synapses from two axons/animals were collected at distances of 30–400 μm surrounding the injection site where protein had diffused based on the visualization of the co-injected fluorescent dye.

For each EM image, the center of the active zone was identified, and a morphometric analysis of synaptic membranes within a 1 μm radius was performed by a researcher blinded to the experimental conditions using FIJI 2.0. As in our previous studies (Medeiros et al., 2017), measurements included the number and diameter of synaptic vesicles per synapse; number and size of large irregularly shaped vesicles (“cisternae”), which are >100 nm in diameter; size of plasma membrane evaginations; and number and stage of clathrin-coated pits (CCPs) and CCVs. Plasma membrane evaginations were measured by drawing a 1 μm straight line from the edge of the active zone to the nearest position on the axolemma, on both sides of the synapse, and then measuring the curved distance between these points. CCPs and CCVs stages (1–4) were defined as described previously (Medeiros et al., 2017). Briefly, stage 1: initiation of clathrin coat formation; stage 2: invagination and maturation of the CCP; stage 3: constriction of the CCP neck and fission; and stage 4: free CCVs separated from the plasma membrane. Additionally, we analyzed the number of atypical vesicular structures that were contiguous with the plasma membrane at the active zone, which we called “fusosomes.” GraphPad 9.0.0 (GraphPad Software, Inc., La Jolla, CA, United States) was used to generate graphs and perform all statistical analyses. Data were reported as the mean value ± SEM per section per synapse. As previously described (Medeiros et al., 2017; Banks et al., 2020; Soll et al., 2020), 3D reconstructions of single synapses from five serial images were generated using Reconstruct software version 1.1.0.0 (Fiala, 2005). Synaptic structures were rendered using trace slabs for the plasma membrane and cisternae; spheres for synaptic vesicles (50 nm) and CCPs and vesicles (90 nm); and a Boissonnat surface for the active zone.

## Results

### α-Synuclein Derived From Normal Human Brains Comprises Helical Multimers

**FIGURE 2:**
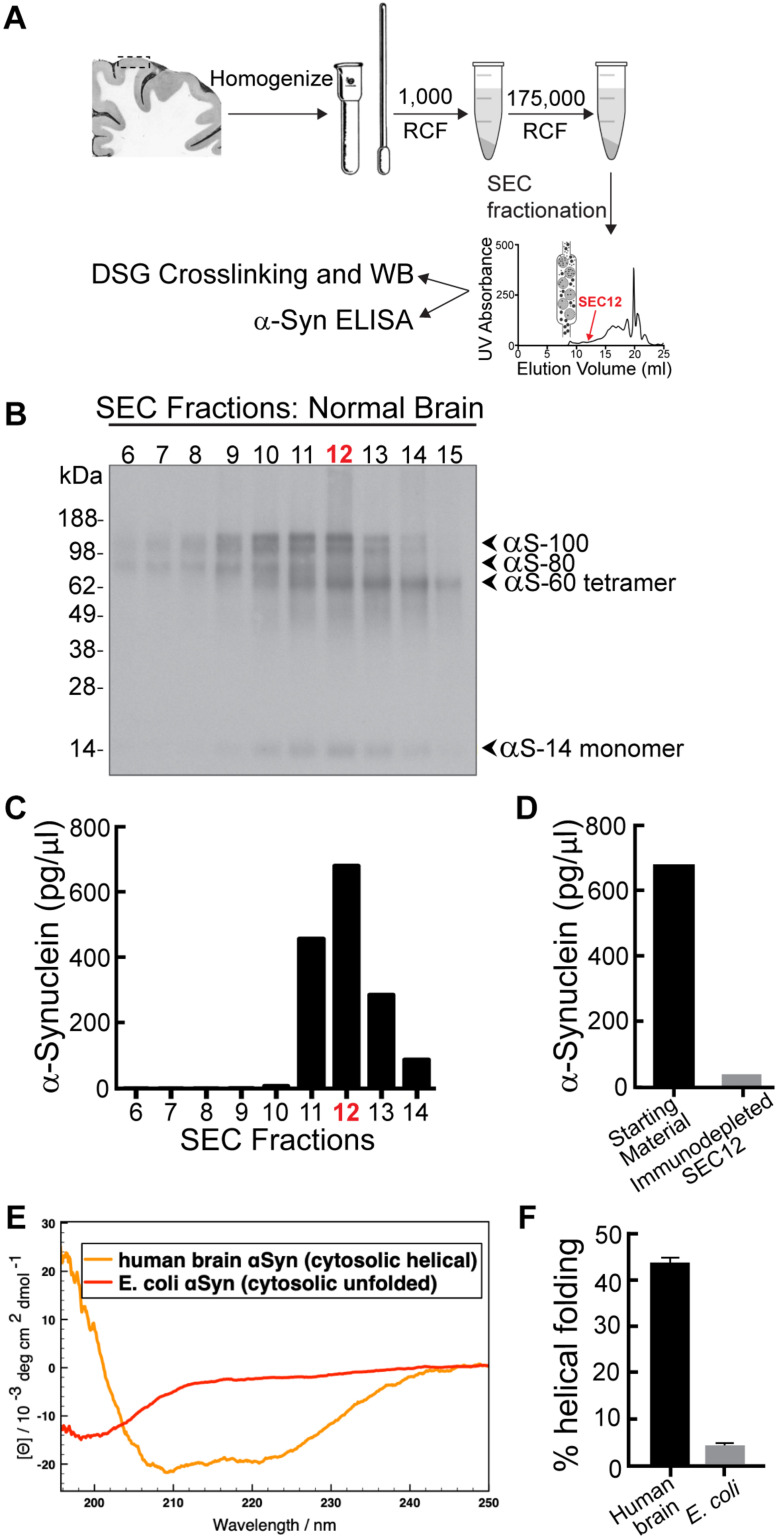
α-Synuclein purified from a neuropathologically normal human brain is predominantly multimeric (60, 80, and 100 kDa species). **(A)** Isolation and purification of α-synuclein from normal human brain. Cortex from a human brain was homogenized and pelleted by a series of centrifugations. Crosslinking with disuccinimidyl glutarate (DSG) crosslinks native multimers in their physiological quaternary structure. **(B)** Western blot showing α-synuclein content in each SEC fraction after DSG crosslinking. Fractions comprise both monomeric α-synuclein (14 kDa) and higher molecular weight α-synuclein multimers (60, 80, and 100 kDa). **(C)** Distribution of α-synuclein in SEC elution fractions, as measured by ELISA. The highest concentration of α-synuclein was in SEC fraction 12 (corresponding to a molecular weight of ∼60–80 kDa). **(D)** Immunodepletion of α-synuclein from fraction 12 was performed using a monoclonal antibody to α-synuclein (clone 2F12, Sigma). α-Synuclein and immunodepleted samples were subsequently microinjected into axons for analysis of synaptic effects. **(E)** Circular dichroism spectroscopy reveals that, unlike *E. coli*-derived α-synuclein, the human brain α-synuclein adopts natively an alpha-helical structure in solution, as shown by the minima of ellipticity at 208 and 220 nm. **(F)** The α-synuclein from human brain exhibits an α-helical secondary structure of ∼45% in solution, while the recombinant protein from *E. coli* is mostly unfolded (∼5% helical fold).

The goal of this study was to determine how increasing the levels of brain-derived human α-synuclein affects synaptic vesicle trafficking. We therefore began by purifying nativeα-synuclein from the brain of a neuropathologically normal human subject, using size exclusion chromatography (SEC) (Figure 2A; see section “Materials and Methods”). Western blot results of cross-linked material showed that the sample comprised a mixed population of α-synuclein species dominated by α-synuclein multimers at 60 (tetramers), 80, and 100 kDa, as well as a minor band of monomeric α-synuclein at 14 kDa (Figure 2B). ELISA analysis showed that SEC fraction 12 had the highest concentration of α-synuclein (Figure 2C; ∼700 pg/μl), and so this fraction was chosen for axonal microinjection. Immunodepletion of human α-synuclein with a monoclonal antibody against α-synuclein (clone 2F12; Sigma-Aldrich) successfully decreased α-synuclein levels by >95%, (Figure 2D), confirming the α-synuclein content and providing a negative control for the synapse experiments. Consistent with prior studies (Bartels et al., 2011; Dettmer et al., 2013; Luth et al., 2015), native α-synuclein multimers derived from normal human brain adopted an alpha-helical secondary structure in solution (Figures 2E,F).

### Brain-Derived Human α-Synuclein Inhibits Synaptic Vesicle Trafficking

To determine whether acute introduction of excess brain-derived human α-synuclein affects synaptic vesicle trafficking, we microinjected α-synuclein-rich SEC fraction 12 (corresponding to a molecular weight of ∼60–80 kDa) into lamprey giant RS axons and determined the subsequent effects on synaptic morphology using standard transmission electron microscopy. The α-synuclein-immunodepleted sample was used as a control. Brain-derived α-synuclein (∼800 nM pipet concentration) was acutely delivered directly to presynaptic terminals via microinjection into lamprey giant axons, as previously described (Figures 1A,B; Busch et al., 2014; Medeiros et al., 2017; Walsh et al., 2018). In order to stimulate exocytosis and endocytosis, axons were subsequently stimulated with 1 ms current pulses to induce action potentials at high frequency (20 Hz, 5 min), immediately fixed and processed for electron microscopy (Figure 1C). Ultrastructural analysis was performed on images of synapses acquired from regions of the axon containing a higher concentration (estimated ∼40–80 nM) versus lower concentration (estimated ∼4–30 nM) of α-synuclein, as well as regions beyond the limit of protein diffusion, which provided an internal control for each experiment (Figure 1A).

**FIGURE 3:**
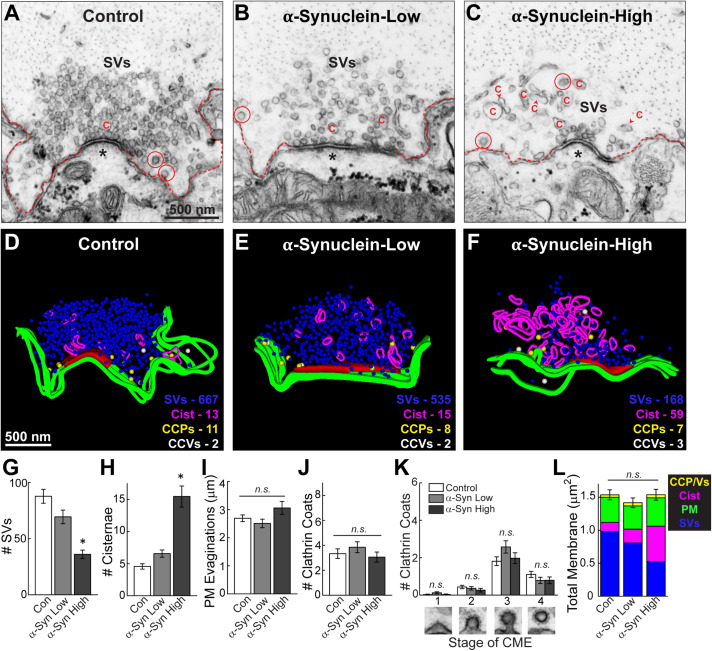
Acute introduction of brain-derived human α-synuclein impairs synaptic vesicle trafficking at lamprey synapses. **(A–C)** Electron micrographs of giant RS synapses showing a stimulated (20 Hz, 5 min) control synapse and those treated with low or high concentrations of brain-derived α-synuclein. Synapses treated with α-synuclein showed a reduction in the number of synaptic vesicles (SVs), and an increase in large, atypical vesicular cisternae “C”. Plasma membrane evaginations (dotted line) and clathrin-coated pits/vesicles (CCP/Vs; circles) were unchanged relative to controls. Asterisks indicate the post-synaptic dendrites. Scale bar in A = 500 nm and applies to B,C. **(D–F)** Three-dimensional reconstructions reveal changes in synapse morphology. SVs are in blue, PM in green, CCPs in yellow, CCVs in white, and cisternae in magenta. Active zone is red. Scale bar in D = 500 nm and applies to E,F. **(G–K)** Quantification of the synaptic vesicle trafficking defect reveals that α-synuclein treated synapses had significantly fewer SVs, which was compensated by an increase in the number of atypical cisternae, and no significant effect on the plasma membrane or CCP/Vs. CME = clathrin-mediated endocytosis. **(L)** A total membrane analysis confirms these compensatory effects. Bars represent mean ± SEM per synapse per section (*n* = 33–37 synapses, two axons/animals). Asterisks indicate statistical significance (*p* < 0.05), and “n.s.” indicates “not significant” by ANOVA.

Control, stimulated lamprey synapses contain large synaptic vesicle clusters, shallow plasma membrane evaginations and only a few CCPs and CCVs (Figures 3A,D). Synapses treated with low concentrations of brain-derived human α-synuclein exhibited slightly fewer synaptic vesicles and more cisternae, defined as any irregular-shaped vesicles with a diameter >100 nm, but no other dramatic changes in morphology compared to control synapses (Figures 3B,E). In comparison, at higher concentrations of α-synuclein, the synaptic vesicle clusters were much smaller, without obvious changes to the plasma membrane or clathrin-coated pit and vesicles (CCP/Vs) (Figures 3C,F). Instead, there was a striking increase in the number of larger cisternae, which are reminiscent of endosomes. 3D reconstructions generated from serial micrographs further emphasized the loss of synaptic vesicles and buildup of large cisternae induced by brain-derived human α-synuclein (Figures 3D–F). In contrast, the plasma membrane evaginations and the number of clathrin coated intermediates did not appear to be affected (Figures 3D–F).

We performed a comprehensive morphometric analysis of all synaptic membranes within 1 μm of the active zone. There was a progressive loss of synaptic vesicles with increasing concentrations of brain-derived human α-synuclein (Figure 3G). Indeed, at higher concentrations, brain-derived α-synuclein caused a statistically significant 59% reduction in the average number of synaptic vesicles, compared to control synapses (Figure 3G; Control: 87.78 ± 6.22 SVs/section, *n* = 37 synapses, two axons; α-Synuclein low: 69.45 ± 6.03 SVs/section, *n* = 33 synapses, two axons; α-Synuclein high: 36.03 ± 3.77 SVs/section, *n* = 34 synapses; two axons; ANOVA; *p* < 0.0001). This loss of synaptic vesicles was compensated by a 3.4-fold increase in the average number of cisternae per synapse (Figure 3H; Control: 4.57 ± 0.41 cisternae, *n* = 37 synapses, two axons; α-Synuclein-Low: 6.73 ± 0.55 cisternae, *n* = 33 synapses, two axons; α-Synuclein-High: 15.47 ± 1.64 cisternae, *n* = 34 synapses, two axons; ANOVA; *p* < 0.0001). In contrast, there was no significant difference in the size of the plasma membrane evaginations (Figure 3I; Control: 2.69 ± 0.12 μm, *n* = 37 synapses, two axons; α-Synuclein-Low: 2.49 ± 0.15 μm, *n* = 33 synapses, two axons; α-Synuclein-High: 3.06 ± 0.23 μm, *n* = 34 synapses, two axons; ANOVA; *p* = 0.0614). Nor were the total numbers of clathrin coated structures (CCPs + CCVs) significantly changed, as compared to controls (Figure 3J; Control: 3.38 ± 0.32 coats, *n* = 37 synapses, two axons; α-Synuclein-Low: 3.85 ± 0.44 coats, *n* = 33 synapses, two axons; α-Synuclein-High: 3.06 ± 0.40 coats, *n* = 34 synapses, two axons; ANOVA; *p* = 0.3602). Brain-derived human α-synuclein also did not appear to affect the progression of clathrin-mediated endocytosis, as shown by the unaltered distributions of CCPs and vesicles (Figure 3K; Stage 1-Control: 0.03 ± 0.03 CCPs; α-Synuclein-Low: 0.12 ± 0.06 CCPs; α-Synuclein-High: 0.03 ± 0.03 CCPs; Stage 2-Control: 0.43 ± 0.09 CCPs; α-Synuclein-Low: 0.36 ± 0.10 CCPs; α-Synuclein-High: 0.26 ± 0.10 CCPs; Stage 3-Control: 1.81 ± 0.24 CCPs; α-Synuclein-Low: 2.58 ± 0.33 CCPs; α-Synuclein-High: 1.97 ± 0.29 CCPs; Stage 4-Control: 1.11 ± 0.16 CCVs; α-Synuclein-Low: 0.79 ± 0.17 CCVs; α-Synuclein-High: 0.79 ± 0.18 CCVs; *n* = 33–37 synapses, two axons per condition; ANOVA *p* = 0.0871; Tukey’s *post hoc*). A total membrane analysis confirmed that the loss of synaptic vesicle membrane corresponded to the increase in cisternae membrane, while the plasma membrane evaginations and clathrin-coated pits and vesicles (CCPs/Vs) remained relatively unchanged (Figure 3L; Control: 1.54 ± 0.08 μm^2^; α-Synuclein-Low: 1.42 ± 0.07 μm^2^; α-Synuclein-High: 1.54 ± 0.08 μm^2^; *n* = 33–37 synapses, two axons/animals per condition; ANOVA *p* = 0.4544). These data indicate that excess brain-derived human α-synuclein, comprising predominantly helical multimers, impacts synaptic vesicles and other vesicular structures at synapses but does not impair clathrin-mediated synaptic vesicle endocytosis from the plasma membrane, consistent with an impairment of intracellular vesicle trafficking.

**FIGURE 4:**
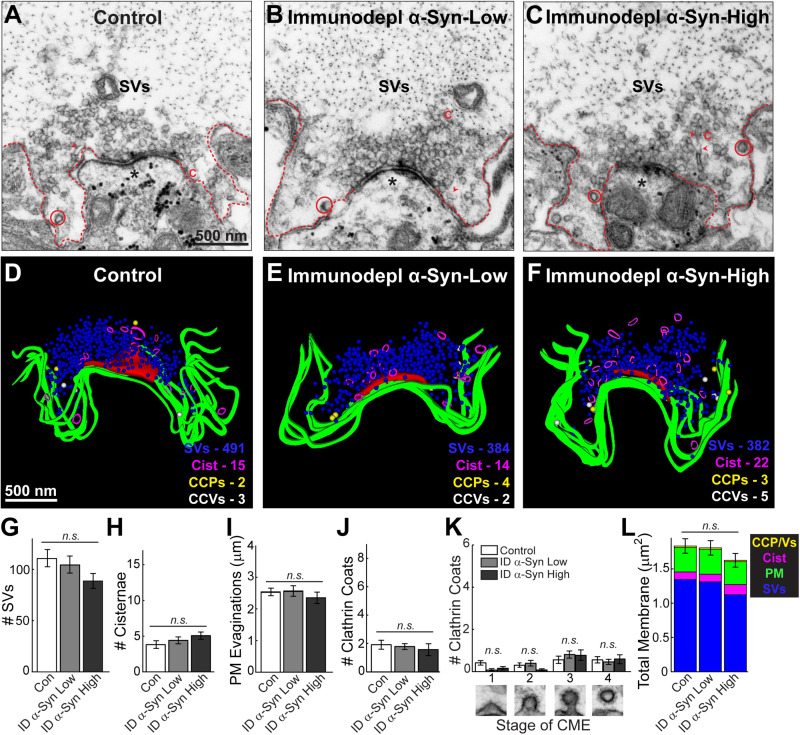
After immunodepletion of α-synuclein, there was no effect on synaptic vesicle trafficking. **(A–C)** Electron micrographs of giant RS synapses showing a stimulated (20 Hz, 5 min) control synapse, as well as synapses treated with low or high concentrations of SEC fraction 12 after immunodepletion of α-synuclein. There was no apparent change in synapse morphology, including the synaptic vesicle (SV) cluster. C = cisternae; dotted lines = plasma membrane (PM); circles = clathrin-coated pits/vesicles (CCP/Vs). Asterisks indicate the post-synaptic dendrites. Scale bar in A = 500 nm and applies to B,C. **(D–F)** Three-dimensional synapse reconstructions. SVs in blue, PM in green, CCP in yellow, CCV in white, and cisternae in magenta. Active zone is in red. Scale bar in D = 500 nm and applies to E,F. **(G–L)** There were no significant changes in synaptic membranes after treatment with a brain sample immunodepleted of α-synuclein, confirming that α-synuclein is required to produce the vesicle trafficking effects shown in Figure 3. Bars represent mean ± SEM per synapse per section (*n* = 21–23 synapses, two axons/animals). ID indicates “immunodepleted.” CME = clathrin-mediated endocytosis. “n.s.” indicates “not significant” by ANOVA.

To confirm that these synaptic vesicle trafficking deficits were caused by α-synuclein and not some other unidentified, co-eluting protein, we also injected axons with a sample that was immunodepleted of α-synuclein. The immunodepleted sample produced no obvious changes in synaptic morphology throughout the axon (Figures 4A–F). Synaptic vesicle clusters and cisternae remained unchanged compared to controls (Figures 4G,H) (Synaptic vesicles, Control: 111.4 ± 8.39 SVs/section, *n* = 21 synapses; Immunodepl α-Synuclein-Low: 105.0 ± 8.36 SVs/section, *n* = 23 synapses; Immunodepl α-Synuclein-High: 89.43 ± 7.76 SVs/section, *n* = 23 synapses; two axons per condition; ANOVA *p* = 0.1444) (Cisternae, Control: 3.86 ± 0.54 cisternae/section, *n* = 21 synapses; Immunodepl α-Synuclein-Low: 4.44 ± 0.49 cisternae/section, *n* = 23 synapses; Immunodepl α-Synuclein-High: 5.09 ± 0.51 cisternae/section, *n* = 23 synapses; two axons per condition; ANOVA; *p* = 0.2496). Likewise, the plasma membrane evaginations were unaltered (Figure 4I; Control: 2.56 ± 0.12 μm, *n* = 21 synapses; Immunodepl α-Synuclein-Low: 2.58 ± 0.16 μm, *n* = 23 synapses; Immunodepl α-Synuclein-High: 2.37 ± 0.18 μm, *n* = 23 synapses; two axons per condition; ANOVA; *p* = 0.5891). The total number of CCPs and vesicles, and their distributions, also remained unchanged (Figures 4J,K) (# Clathrin coats, Control: 1.95 ± 0.32 coats, *n* = 21 synapses; Immunodepl α-Synuclein-Low: 1.83 ± 0.21 coats, *n* = 23 synapses; Immunodepl α-Synuclein-High: 1.61 ± 0.42 coats, *n* = 23 synapses; two axons per condition; ANOVA; *p* = 0.7576) (Stage 1-Control: 0.43 ± 0.11 CCPs; Immunodepl α-Synuclein-Low: 0.09 ± 0.06 CCPs; Immunodepl α-Synuclein-High: 0.17 ± 0.08 CCPs; Stage 2-Control: 0.33 ± 0.11 CCPs; Immunodepl α-Synuclein-Low: 0.43 ± 0.14 CCPs; Immunodepl α-Synuclein-High: 0.09 ± 0.06 CCPs; Stage 3-Control: 0.57 ± 0.18 CCPs; Immunodepl α-Synuclein-Low: 0.83 ± 0.17 CCPs; Immunodepl α-Synuclein-High: 0.78 ± 0.25 CCPs; Stage 4-Control: 0.57 ± 0.15 CCVs; Immunodepl α-Synuclein-Low: 0.48 ± 0.12 CCVs; Immunodepl α-Synuclein-High: 0.61 ± 0.22 CCVs; *n* = 21–23 synapses, two axons per condition; ANOVA *p* = 0.2920; Tukey’s *post hoc*). Finally, a total membrane analysis showed no significant changes in membrane distribution (Figure 4L; Control: 1.84 ± 0.10 μm^2^; Immunodepl α-Synuclein-Low: 1.80 ± 0.10 μm^2^; Immunodepl α-Synuclein-High: 1.63 ± 0.10 μm^2^, *n* = 21–23 synapses, two axons per condition; ANOVA; *p* = 0.3081). Together, these results show that the synaptic vesicle trafficking defects observed were specifically due to the presence of α-synuclein, as immunodepletion of α-synuclein eliminated these morphological effects.

### Brain-Derived Human α-Synuclein Affects Vesicle Morphology

**FIGURE 5:**
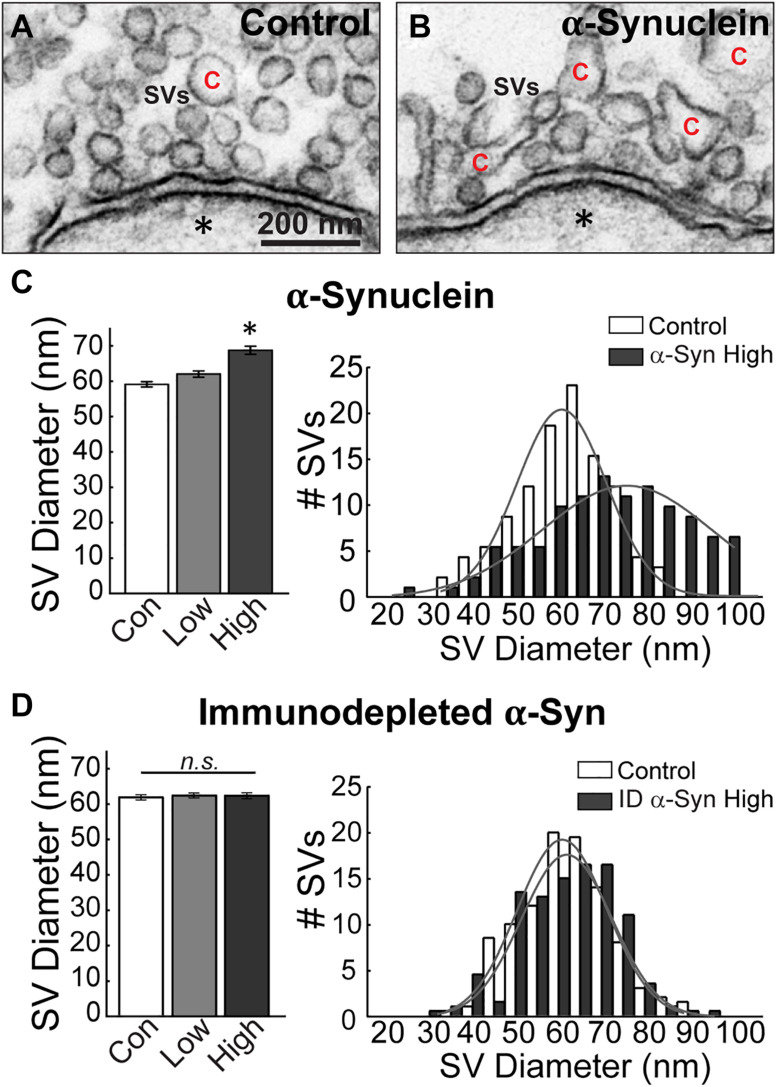
Excess brain-derived human α-synuclein leads to increased synaptic vesicle diameter. **(A,B)** Electron micrographs showing synaptic vesicles (SVs) in a control synapse and a synapse treated with brain-derived human α-synuclein (high concentration). α-Synuclein causes SVs to become larger and more heterogeneous in size. “C” = cisternae. Scale bar in A = 200 nm and applies to B. **(C,D)** Quantification revealed that SVs had increased diameters and heterogeneous distribution after treatment with α-synuclein (*n* = 34–37 synapses, two axons/animals), but not after immunodepletion of α-synuclein (*n* = 21–23 synapses), two axons/animals. Bars represent mean ± SEM per synapse per section. “ID” indicates “immunodepleted.” Asterisks indicate statistical significance (*p* < 0.05), and “n.s.” indicates “not significant” by ANOVA.

To further investigate the alterations in the vesicular structures, we performed additional analyses on the synaptic vesicle sizes and distributions. Synaptic vesicles were defined as small, clear round vesicles that were homogeneous in size, typically around ∼50–60 nm in diameter (but under 100 nm). Compared to synaptic vesicles at untreated control synapses (Figure 5A), those treated with excess brain-derived human α-synuclein appeared larger and more heterogenous in size (Figure 5B). Quantitative analysis showed that excess α-synuclein caused a statistically significant 16.22% increase in the mean synaptic vesicle diameter at the highest concentration, compared to control (Figure 5C, left; Control, 59.68 ± 0.76 nm, *n* = 37 synapses, two axons; α-Synuclein-Low: 62.60 ± 0.86 nm, *n* = 33 synapses; two axons; α-Synuclein-High: 69.36 ± 1.18 nm, *n* = 34 synapses; two axons; ANOVA; *p* < 0.0001; Tukey’s *post hoc*). Synaptic vesicle size distribution revealed a rightward shift in the peak with brain-derived human α-synuclein (Figure 5C, right; mean SV diameter: control, 62.29 nm; α-Synuclein-High, 72.06 nm; two-tailed test *p* < 0.0001). Confirming that these effects were due to α-synuclein, the mean synaptic vesicle diameter after α-synuclein immunodepletion was unchanged compared to control (Figure 5D, left; Control: 61.91 ± 0.75 μm, *n* = 21 synapses; ID α-Synuclein-Low: 62.70 ± 0.73 μm, *n* = 23 synapses; ID α-Synuclein-High: 62.59 ± 0.84 μm, *n* = 23 synapses; two axons; ANOVA; *p* = 0.7724; Tukey’s *post hoc*). Moreover, the size distribution was also unchanged (Figure 5D, right; mean SV diameter: control, 61.91 nm; ID α-Synuclein, high, 62.59 nm; two-tailed *t*-test *p* = 0.5843).

**FIGURE 6:**
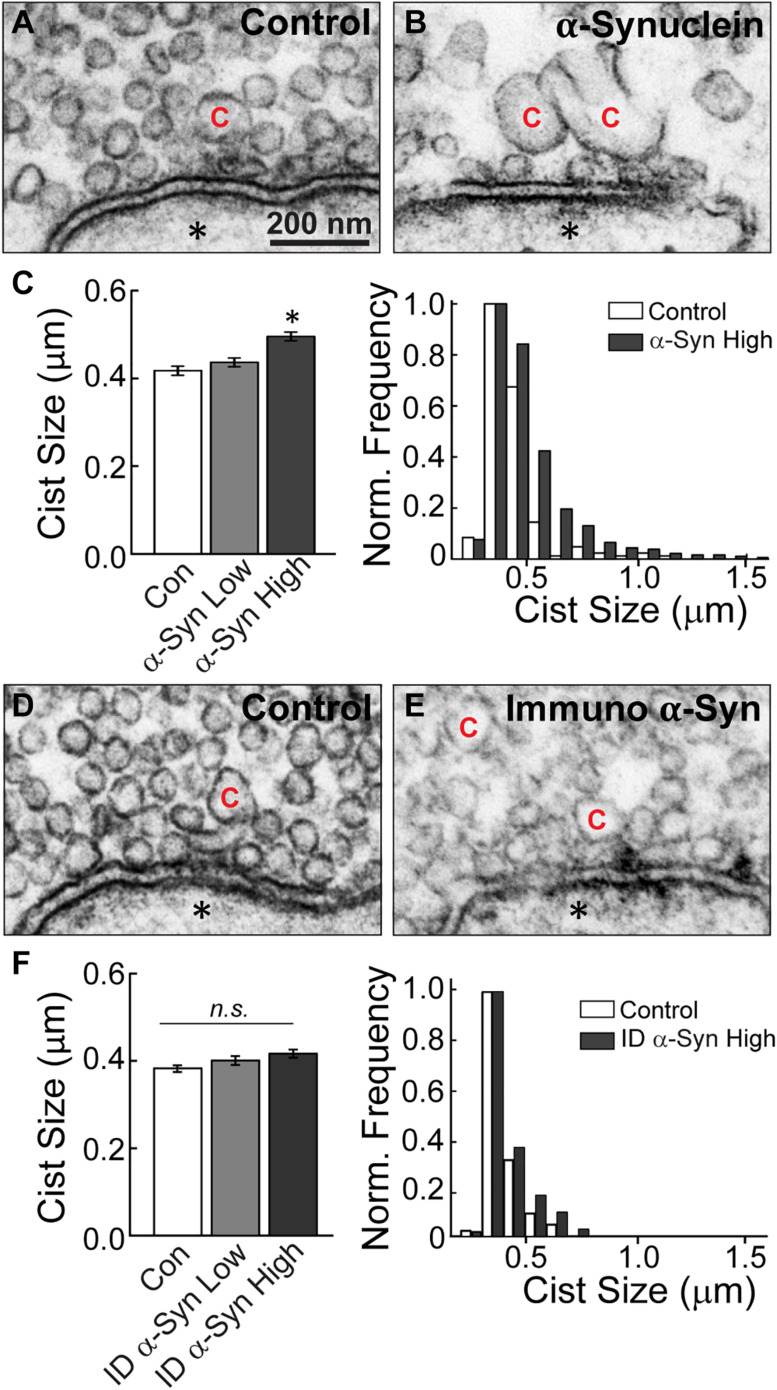
Excess brain-derived human α-synuclein induces the appearance of large cisternae. **(A,B)** Electron micrographs of cisternae “C” observed at control synapses or after treatment with excess brain-derived α-synuclein. α-Synuclein induces larger cisternae. Asterisks indicate the post-synaptic dendrites. Scale bar in A = 200 nm and applies to B,D,E. **(C)** Quantification of cisternae measurements from α-synuclein treated synapses reveal an increase in the average size of cisternae (left) and expanded distribution (right) (*n* = 139–536 cisternae, 33–37 synapses, two axons). **(D,E)** Micrographs of a control synapse and a synapse treated with the α-synuclein immunodepleted sample. **(F)** After immunodepletion of α-synuclein, synapses show no significant alterations in average cisternae sizes (left) or distribution (right) (*n* = 83–119 cisternae, 21–23 synapses, two axons). Bars represent mean ± SEM per synapse per section. “ID” indicates “immunodepleted.” Asterisks indicate statistical significance (*p* < 0.05), and “n.s.” indicates “not significant” by ANOVA.

Next, we performed analyses on the size and distribution of the cisternae, which were also altered after treatment with brain-derived human α-synuclein (see Figure 3). Cisternae were defined as irregular-shaped vesicles that were greater than 100 nm in diameter. Morphological analysis of the cisternae revealed that those induced by α-synuclein were also larger than those at control synapses (Figures 6A,B). The vast majority (∼98%) of the cisternae were large, disconnected from the plasma membrane, and devoid of budding CCPs (Figure 6B). The remaining ∼2% of cisternae exhibited CCPs emanating from them and trended toward larger sizes (Supplementary Figure 1). Quantitative analysis revealed an increase in the average cisternae size at α-synuclein treated synapses, as measured by the perimeter of these vesicular structures (Figure 6C, left; Control, 0.43 ± 0.01 μm, *n* = 139 cisternae, 37 synapses; ANOVA; α-Synuclein-Low: 0.45 ± 0.01 μm, *n* = 220 cisternae, 33 synapses; two axons; α-Synuclein-High: 0.53 ± 0.02 μm, *n* = 536 cisternae, 34 synapses; two axons; ANOVA; *p* = 0.0009). However, there was no significant change in the peak size for the cisternae (Figure 6C, right; peak cisternae size: Control, 0.39 ± 0.07 μm; α-Synuclein-High: 0.41 ± 0.09 μm; two-tailed *t*-test *p* = 0.6859). Confirming that these effects were specifically due to α-synuclein, synapses treated with the α-synuclein-immunodepleted sample showed no significant change in the mean cisternae size compared to controls (Figures 6D–F, left; Control: 0.39 ± 0.01 μm, *n* = 83 cisternae, 21 synapses; Immunodepl α-Synuclein-Low: 0.40 ± 0.01 μm, *n* = 100 cisternae, 23 synapses; Immunodepl α-Synuclein-High: 0.42 ± 0.01 μm, *n* = 119 cisternae, 23 synapses; two axons; ANOVA; *p* = 0.0984). Nor was the cisternae size distribution altered (Figure 6F, right; peak cisternae size: Control, 0.38 ± 0.04 μm; Immunodepl α-Synuclein-High, 0.38 ± 0.05 μm; two-tailed *t*-test *p* = 0.8749). Taken together, these data indicate that excess brain-derived human α-synuclein affects synaptic vesicle and cisternae morphology, which is consistent with an impairment in intracellular vesicle trafficking.

### Brain-Derived Human α-Synuclein Induces Atypical Fusion/Fission Events at the Active Zone

**FIGURE 7:**
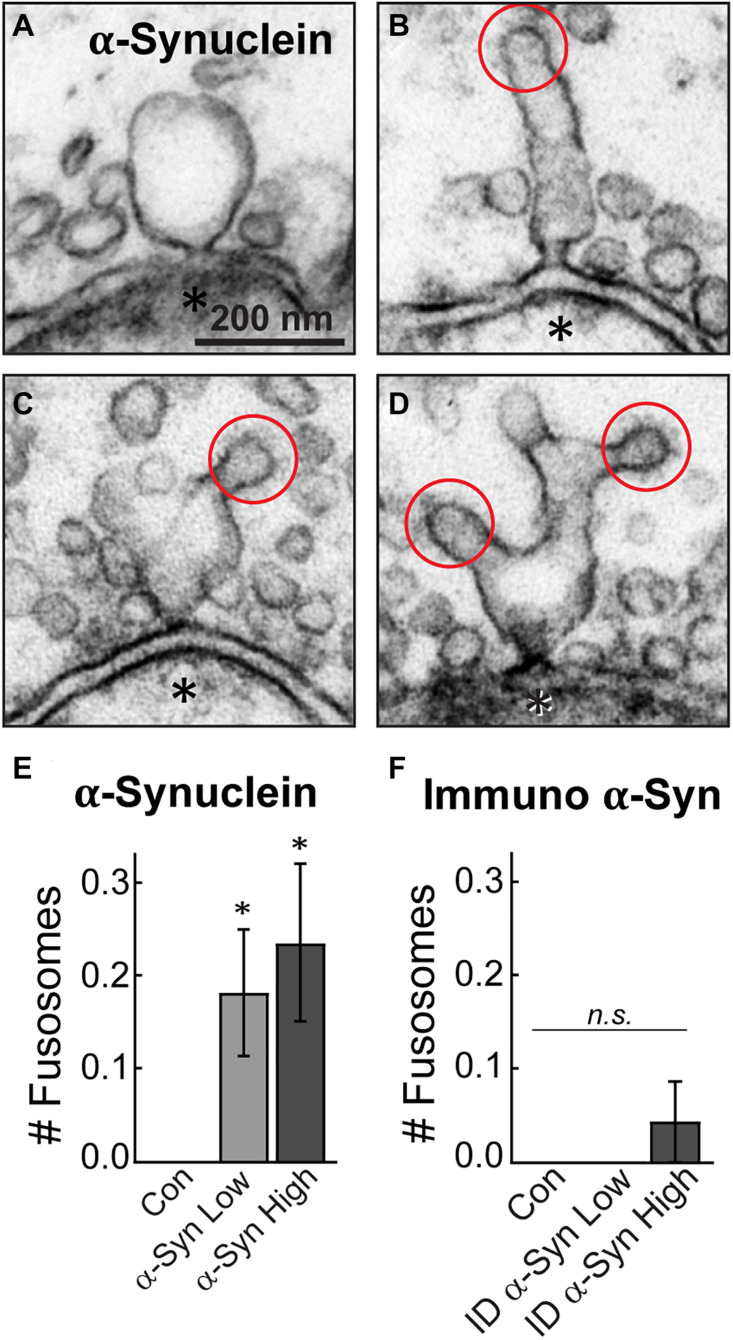
Excess brain-derived human α-synuclein produces abnormal fusion/fission events. **(A–D)** Electron micrographs showing morphology of “fusosomes” in the active zone induced by excess α-synuclein. Red circles indicate CCPs emanating from some fusosomes. Asterisks indicate post-synaptic dendrites. Scale bar in A = 200 nm and applies to B–D. **(E,F)** Quantification reveals a significant increase in the number of fusosomes per synapse after treatment with α-synuclein (*n* = 34–37 synapses, two axons), but not with the immunodepleted sample (*n* = 21–23 synapses, two axons). Data represent mean ± SEM per synapse per section. “ID” indicates “immunodepleted.” Asterisks indicate statistical significance (*p* < 0.05), and “n.s.” indicates “not significant” by ANOVA.

Finally, a striking phenotype induced by brain-derived human α-synuclein was the appearance of large, atypical vesicular structures at the active zone, which were fused and contiguous with the plasma membrane (Figure 7). These structures, which we have termed “fusosomes,” appeared to have stiff necks connecting them to the plasma membrane. Some looked like cisternae attached to the plasma membrane (Figure 7A), while others appeared as multivesicular tubules or large cisternae with CCPs budding from them (Figures 7B–D). These structures were completely absent from untreated, control synapses. Compared to controls, brain-derived α-synuclein increased the mean number of fusosomes at synapses (Figure 7E; Control: 0 ± 0 fusosome/synapse; *n* = 49 synapses; two axons; α-Synuclein-Low: 0.18 ± 0.07 fusosomes/synapse; *n* = 34; two axons; α-Synuclein-High: 0.24 ± 0.09 fusosomes/synapse; *n* = 34; two axons; ANOVA, *p* = 0.0049). These structures were markedly reduced after α-synuclein immunodepletion (Figure 7F; Control: 0 ± 0 fusosome/synapse; *n* = 21 synapses; Immunodepl α-Synuclein-Low: 0 ± 0 fusosomes/synapse; *n* = 23; Immunodepl α-Synuclein-High: 0.04 ± 0.04 fusosomes/synapse; *n* = 23; two axons; ANOVA, *p* = 0.9552). Furthermore, when we reinvestigated our data from a prior study (Medeiros et al., 2017), we never observed fusosomes after introduction of excess recombinant monomeric or two forms of dimeric human α-synuclein, even at much higher concentrations ranging from 50 to 160 μM (Control: 0 ± 0 fusosome/synapse; monomeric α-synuclein: 0 ± 0 fusosome/synapse; dimeric α-synuclein-CC: 0 ± 0 fusosome/synapse; dimeric α-synuclein-NC: 0 ± 0 fusosome/synapse; *n* = 21–39 synapses; two axons/animals). Thus, introduction of excess α-synuclein derived from a normal human brain uniquely induced atypical fusion/fission events at synaptic active zones.

## Discussion

**FIGURE 8:**
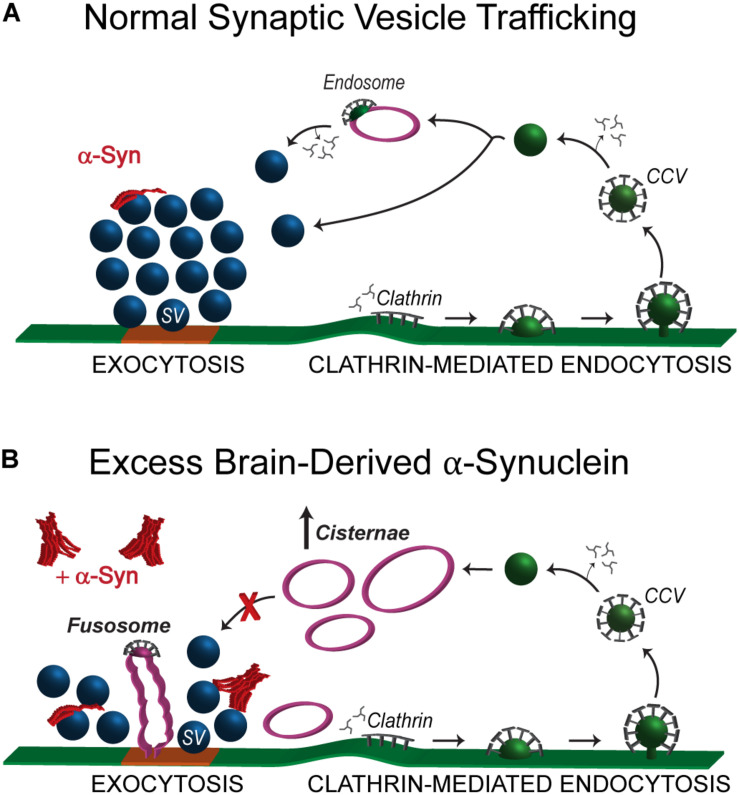
Excess brain-derived human α-synuclein produces intracellular trafficking defects without effects on clathrin mediated synaptic vesicle endocytosis. **(A)** Model showing normal synaptic vesicle trafficking with emphasis on clathrin-mediated endocytosis as the pathway for recycling vesicles. Under normal conditions, α-synuclein localizes to presynaptic terminals where it interacts with the synaptic vesicle cluster and regulates both exocytosis and endocytosis. **(B)** Acute introduction of brain-derived human α-synuclein, comprising predominantly helical multimers of α-synuclein, alters intracellular vesicle trafficking as characterized by the reduced number of synaptic vesicles, an increase in the number of aberrant cisternae and the appearance of fusosomes at the active zone. The working hypothesis is that the cisternae are likely to be recycling and/or bulk endosomes.

We report here that acute introduction of excess native α-synuclein purified from a neuropathologically normal human brain induced moderate vesicle trafficking defects at synapses. This was evidenced by a reduction in the number of synaptic vesicles along with a compensatory increase in the number and size of atypical cisternae (Figures 3, 6) and larger synaptic vesicles (Figure 5). In contrast, the plasma membrane evaginations and clathrin-coated intermediates were relatively normal (Figure 3). These data indicate that clathrin-mediated synaptic vesicle endocytosis from the plasma membrane was relatively unaffected after introduction of excess brain-derived human α-synuclein and suggest that instead there was a moderate impairment of intracellular vesicle trafficking (Figure 8). The phenotypes observed can likely be attributed to the native α-synuclein multimers (e.g., tetramers and related multimers), since these are the predominant species in the purified brain sample.

The phenotype produced by brain-derived human α-synuclein was strikingly different than the phenotypes produced by recombinant monomeric and dimeric human α-synuclein, which we previously reported (Busch et al., 2014; Medeiros et al., 2017; Banks et al., 2020). In addition to the loss of synaptic vesicles and increased numbers of cisternae, monomeric and dimeric α-synuclein also induced a dramatic expansion of the plasma membrane and increased numbers of CCPs and CCVs, consistent with an inhibition of clathrin-mediated synaptic vesicle endocytosis from the plasma membrane (Medeiros et al., 2017, 2018; Banks et al., 2020; Soll et al., 2020). In contrast, brain-derived human α-synuclein did not appear to affect synaptic vesicle endocytosis from the plasma membrane, as the effects on plasma membrane evaginations and clathrin-mediated intermediates were absent when this sample was introduced (Figures 3, 8). This may imply that the presence of native multimers, or some other feature of brain-derived human α-synuclein (e.g., post-translational modifications), may be selectively protective against the endocytic defects caused by monomeric α-synuclein, even though deficits in other intracellular vesicle trafficking steps persisted. However, we acknowledge the difficulty of comparing results between recombinant α-synuclein versus brain-derived material, as the brain-derived human α-synuclein was injected at much lower concentrations due to the limited starting material. That said, even at lower nanomolar concentrations of recombinant dimeric α-synuclein, we observed dramatic impacts on the budding of CCPs from the plasma membrane (Medeiros et al., 2017). So, while we still do not fully understand the specific effects of each different α-synuclein species (i.e., 14, 60, 80, and 100 kDa) within this brain-derived sample, this study does provide further corroborating evidence that different molecular species of α-synuclein produce distinct morphological effects at synapses (Medeiros et al., 2018; Banks et al., 2020; Soll et al., 2020).

So far, a recurrent phenotype observed with all of the molecular species of α-synuclein that we have studied is the increase in the number of atypical cisternae. Similar to our results, enlargement of vesicles is evident after α-synuclein overexpression at mammalian hippocampal synapses (Scott et al., 2010) and in a *Drosophila* model of α-synuclein overexpression (Breda et al., 2015). We have previously suggested that the cisternae produced by monomeric α-synuclein are a result of impaired endocytosis (Busch et al., 2014), and that they may have a role in restoring the synaptic vesicle pool when the α-synuclein-induced trafficking defects were rescued by exogenous Hsc70 (Banks et al., 2020). However, the identity and function of cisternae induced by different α-synuclein species, including those produced by the brain-derived sample, are still unknown. Our results show that the majority of cisternae induced by brain-derived human α-synuclein are disconnected from the plasma membrane, suggesting that they may be recycling endosomes, or alternatively bulk endosomes (Figure 8; Chanaday et al., 2019). However, considering that less than 2% of the cisternae had clathrin-coated buds, it is also plausible that brain-derived human α-synuclein induces the formation of cisternae through aberrant vesicle-vesicle fusion, which has been reported for high concentrations of α-synuclein *in vitro* (Varkey et al., 2010; Fusco et al., 2016; Man et al., 2020). We think this is unlikely, however, because native α-synuclein does not affect v-/t-SNARE-mediated vesicle fusion at lower concentrations closer to those injected in our experiments (Diao et al., 2013).

Another intriguing finding from this study is the appearance of atypical fusion/fission events at the active zones of synapses, which we termed “fusosomes” (Figure 7). We have not previously observed this phenotype in any of our studies focusing on other α-synuclein species. Although we do not yet know the precise identity of these structures, some are reminiscent of compound exocytosis events, which have been observed in ribbon synapses, pituitary lactotrophs, and other neuroendocrine cells (Cochilla et al., 2000; Matthews and Sterling, 2008; Vardjan et al., 2013). This may suggest that brain-derived human α-synuclein promotes compound fusion *in vivo*. In support of this idea, several recent studies have reported that α-synuclein induces multivesicular assemblies, leading to tubulation and vesicle-vesicle fusion *in vitro*, although only at higher concentrations (Varkey et al., 2010; Fusco et al., 2016; Man et al., 2020). Adding to the complex nature of this phenotype, the fusosomes have characteristics of both fusion and fission events (Shin et al., 2018). In support of a possible effect on fusion, α-synuclein overexpression has been shown to promote dilation of the exocytic fusion pore (Logan et al., 2017). However, the stiff neck observed on fusosomes is also characteristic of clathrin-mediated endocytic fission events (Shupliakov et al., 1997; Gad et al., 2000; Sundborger et al., 2011). Whatever their precise identities, current data are consistent with these structures ultimately originating from synaptic vesicles.

Taken together, the predominant effects of brain-derived human α-synuclein seemed to be on highly curved vesicular structures including synaptic vesicles, cisternae, and fusosomes, but not on the plasma membrane. It is well established that recombinant α-synuclein binds to high curvature membranes and that it can induce membrane curvature (Davidson et al., 1998; Burre et al., 2010; Pranke et al., 2011; Westphal and Chandra, 2013). We and others have shown that native α-synuclein, including from human brain (Figures 2D,E; Bartels et al., 2011; Burré et al., 2014; Gould et al., 2014; Kim et al., 2018), displays alpha-helical structure natively and when in contact with small lipid vesicles (Bartels et al., 2011; Diao et al., 2013). Our ultrastructural analyses would therefore suggest that helically folded brain-derived human α-synuclein preferentially interacts with lipid or protein components of vesicles, such as phosphatidyl-inositol-4-phosphate [PI(4)P] which is enriched in synaptic vesicles (Di Paolo and De Camilli, 2006), or the synaptic vesicle-associated membrane protein 2 (VAMP2), which is a well-established α-synuclein interactor (Diao et al., 2013; Burré et al., 2014; Burré et al., 2018). Indeed, a prior mass spectrometry analysis revealed increased binding of oligomeric α-synuclein to VAMP2, synapsin, and several other synaptic vesicle-associated proteins, relative to monomeric α-synuclein (Betzer et al., 2015). Although we still do not know the mechanism by which brain-derived human α-synuclein alters vesicle morphologies, leading to larger synaptic vesicles, appearance of cisternae, and aberrant fusosomes, it is likely that these enhanced vesicle interactions play a major role. α-Synuclein also exhibits enhanced binding to the clathrin assembly protein AP180 upon synaptic stimulation (Vargas et al., 2014). Thus, another possibility is that introducing an excess of brain-derived human α-synuclein may interfere with AP180 function, which is known to lead to increased vesicle diameters and appearance of cisternae (Zhang et al., 1998; Morgan et al., 1999), though in this case effects on clathrin-mediated endocytosis from the plasma membrane would be expected.

In summary, although brain-derived human α-synuclein impaired vesicle trafficking at synapses, there were no obvious effects on synaptic vesicle endocytosis from the plasma membrane. This stands in stark contrast to synaptic phenotypes produced bypurified monomeric or dimeric α-synuclein, which impaired clathrin-mediated synaptic vesicle endocytosis and led to agreater 70–90% depletion of the synaptic vesicle cluster coincidentwith an expanded plasma membrane (Busch et al., 2014; Medeiros et al., 2017; Banks et al., 2020; Soll et al., 2020). This suggests the interestingpossibility that native α-synuclein multimers (e.g., tetramers and related multimers) may be at least partially protectiveagainst the endocytic defects caused by monomericα-synuclein at synapses. Supporting this idea, pointmutations that destabilize tetramers and lead to increased monomericα-synuclein induce aggregation and cause greatertoxicity in cell lines and animal models (Dettmer et al., 2015a, b, 2017). Going forward, it will be important to continue examining thecellular and synaptic effects of different purified forms ofα-synuclein multimers. In addition, since these datawere obtained with α-synuclein purified from thebrain of a single individual, it will also be important to compareresults from multiple individuals, both normal and diseased, in orderto understand the phenotypic consistency and variability and to fully assess the impacts of various forms of α-synuclein on synapses. Understanding how different molecular species of α-synuclein affect synaptic vesicle trafficking remains a key step toward elucidating the pathological mechanisms that lead to synaptic dysfunction and clinically significant neurodegeneration.

## Data Availability Statement

The raw data supporting the conclusions of this article will be made available by the authors, without undue reservation.

## Ethics Statement

The animal study was reviewed and approved by Institutional Animal Care and Use Committee at the Marine Biological Laboratory in Woods Hole, MA, United States and following standards set by the National Institutes of Health.

## Author Contributions

All authors made substantial contributions to the conception and design of the study, were involved in drafting this manuscript, have provided final approval of this manuscript for submission, and agreed to be accountable for all aspects of the work. CR-V, AM, JS, and HJ: data acquisition. CR-V, AM, and JM: data analysis. CR-V, AM, JS, HJ, TB, and JM: interpretation. JS, HJ, and TB: generated and characterized the human α-synuclein and immunodepleted samples, which were critical reagents that were essential for the study.

## Conflict of Interest

The authors declare that the research was conducted in the absence of any commercial or financial relationships that could be construed as a potential conflict of interest.

## Supplementary Material

Supplementary Figure 1 **(A)** Electron micrograph of a large cistern (“C”) with a budding clathrin-coated pit (CCP) (circle) observed in high concentrations of brain-derived human α-synuclein. **(B)** Percentage of cisternae with CCPs from all analyzed synapses, which comprised only ∼2% of the total population. **(C,D)** Graphs comparing the sizes of cisterna without and with CCPs in low versus high concentrations of brain-derived human α-synuclein. [α-Synuclein-Low (−CCPs): 0.44 ± 0.01 μm, *n* = 215 cisternae, 33 synapses; (+CCPs): 0.62 ± 0.07 μm, *n* = 5 cisternae, 33 synapses; Asterisk indicates *p* < 0.05 by Students *t*-test] [α-Synuclein-High (−CCPs): 0.52 ± 0.02 μm; *n* = 526 cisternae, 34 synapses; (+CCPs): 0.76 ± 0.09 μm; *n* = 10 cisternae, 34 synapses, “n.s.” indicates “not significant” by Student’s *t*-test].
