# Supplementary figures and images for: Effects of Excess Brain-Derived Human α-Synuclein on Synaptic Vesicle Trafficking
Source: Front Neurosci. 2021 Feb 4;15:639414. doi: 10.3389/fnins.2021.639414 (PMC7890186; doi:10.3389/fnins.2021.639414)

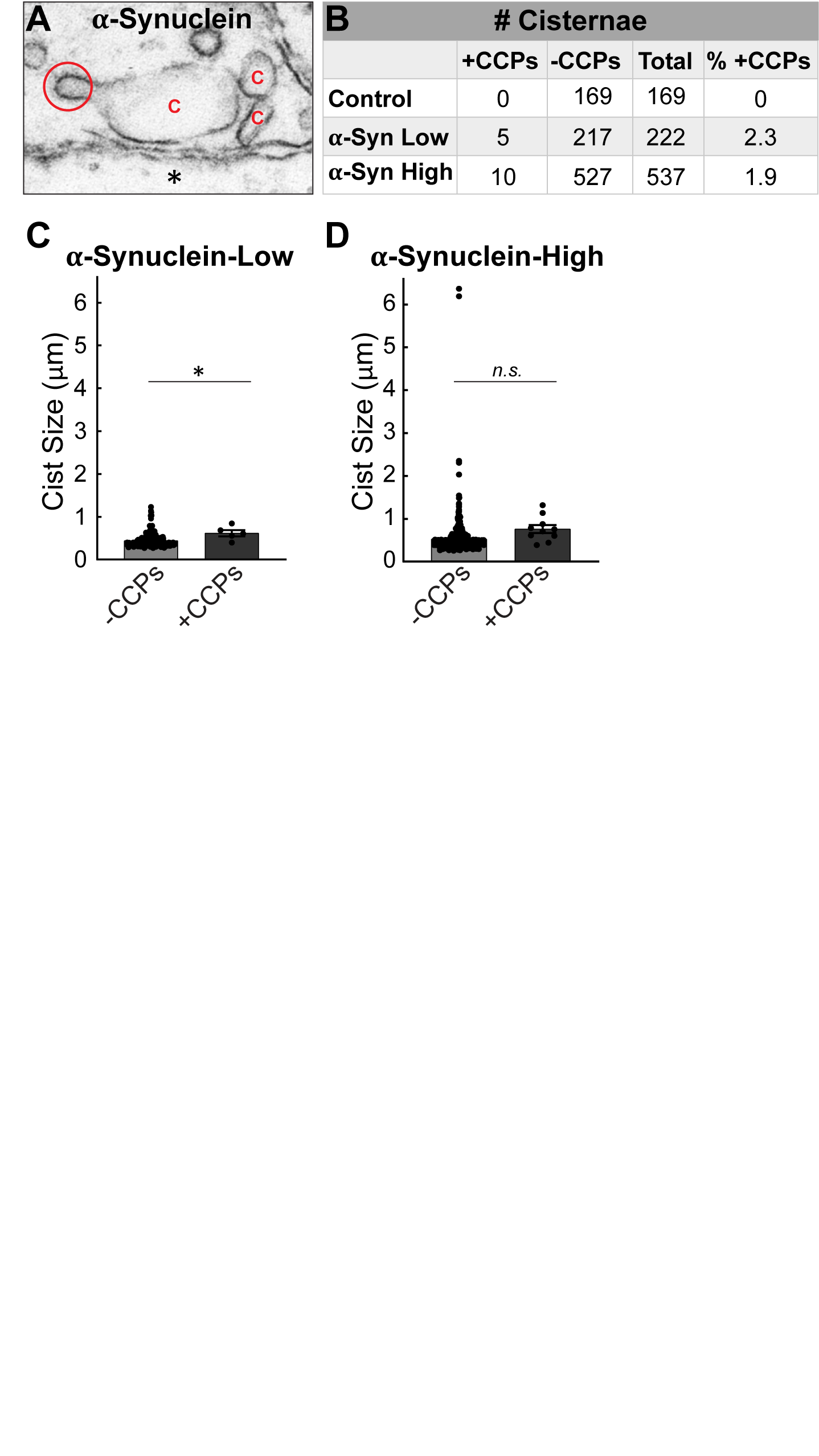

Supplement: Supplementary Figure 1 — (A) Electron micrograph of a large cistern (“C”) with a budding clathrin-coated pit (CCP) (circle) observed in high concentrations of brain-derived human α-synuclein. (B) Percentage of cisternae with CCPs from all analyzed synapses, which comprised only ∼2% of the total population. (C,D) Graphs comparing the sizes of cisterna without and with CCPs in low versus high concentrations of brain-derived human α-synuclein. [α-Synuclein-Low (−CCPs): 0.44 ± 0.01 μm, n = 215 cisternae, 33 synapses; (+CCPs): 0.62 ± 0.07 μm, n = 5 cisternae, 33 synapses; Asterisk indicates p < 0.05 by Students t-test] [α-Synuclein-High (−CCPs): 0.52 ± 0.02 μm; n = 526 cisternae, 34 synapses; (+CCPs): 0.76 ± 0.09 μm; n = 10 cisternae, 34 synapses, “n.s.” indicates “not significant” by Student’s t-test]. [file Image_1.TIF]
